# Application of Neuromorphic Olfactory Approach for High-Accuracy Classification of Malts

**DOI:** 10.3390/s22020440

**Published:** 2022-01-07

**Authors:** Anup Vanarse, Adam Osseiran, Alexander Rassau, Peter van der Made

**Affiliations:** 1 Brainchip Research Institute, Perth 6000, Australia; aosseiran@brainchip.com (A.O.); pmade@brainchip.com (P.v.d.M.); 2 School of Engineering, Edith Cowan University, Joondalup 6027, Australia; a.rassau@ecu.edu.au

**Keywords:** neuromorphic olfaction, bioinspired olfaction, artificial olfactory systems, electronic nose systems, neuromorphic engineering, spiking neural networks

## Abstract

Current developments in artificial olfactory systems, also known as electronic nose (e-nose) systems, have benefited from advanced machine learning techniques that have significantly improved the conditioning and processing of multivariate feature-rich sensor data. These advancements are complemented by the application of bioinspired algorithms and architectures based on findings from neurophysiological studies focusing on the biological olfactory pathway. The application of spiking neural networks (SNNs), and concepts from neuromorphic engineering in general, are one of the key factors that has led to the design and development of efficient bioinspired e-nose systems. However, only a limited number of studies have focused on deploying these models on a natively event-driven hardware platform that exploits the benefits of neuromorphic implementation, such as ultra-low-power consumption and real-time processing, for simplified integration in a portable e-nose system. In this paper, we extend our previously reported neuromorphic encoding and classification approach to a real-world dataset that consists of sensor responses from a commercial e-nose system when exposed to eight different types of malts. We show that the proposed SNN-based classifier was able to deliver 97% accurate classification results at a maximum latency of 0.4 ms per inference with a power consumption of less than 1 mW when deployed on neuromorphic hardware. One of the key advantages of the proposed neuromorphic architecture is that the entire functionality, including pre-processing, event encoding, and classification, can be mapped on the neuromorphic system-on-a-chip (NSoC) to develop power-efficient and highly-accurate real-time e-nose systems.

## 1. Introduction

Research in machine olfaction and electronic nose (e-nose) systems has garnered much interest due to a number of novel applications that can be envisaged by implementing this technology [1]. Although foundational work in odor sensing can be traced back to the 1960s starting with Moncrieff’s mechanical model [2], a paradigm shift in this domain came after the seminal work of Persaud and Dodd [3] in the early 1980s that sparked the development of sophisticated e-nose systems. Inspired by the biological olfactory pathway, Persaud and Dodd proposed an electronic nose system that implemented a multi-sensor approach, combined with a signal conditioning and processing module, for the identification of various volatile compounds. The past thirty years have seen an increasingly large number of studies building on this foundational research to link the functional emulation of the biological olfactory pathway to artificial olfactory systems that can be implemented for real-world applications [1,4,5,6].

Typically comprising a sensor array and a pattern recognition engine (PARC), e-nose systems mimic the capabilities of biological olfaction to recognize chemical analytes. A conventional approach of processing electronic nose data includes four key stages: data acquisition of time-series resistance data generated by the front-end sensing array; application of pre-processing or signal conditioning techniques for denoising; feature extraction of robust information to enhance class differentiability; and a subsequent pattern recognition algorithm that can classify the extracted features to identify the odor class. Although the dynamics of all the aforementioned processes are vital for the implementation of a robust and reliable e-nose system, the PARC engine, in particular, is a principal determining factor for key performance parameters such as power and computing requirements, portability, and classification latency and accuracy [7,8]. The implementation of traditional computing techniques has imposed limitations in handling continuous multi-dimensional data, which in turn has affected the efficiency of the e-nose systems and impeded their performance [4].

Advanced research in machine learning and statistical algorithms has been a major enabler to improved handling of multivariate data, which has led to novel algorithms being implemented for pattern recognition in e-nose systems [4,6,9,10,11]. However, the efficiency of these algorithms has largely depended on pre-processing methods such as dimensionality reduction, and a number of signal conditioning stages that has added to the complexity, power and computational requirements, and the overall processing latency [1,12]. Nevertheless, the limitations observed in these implementations has highlighted the importance of a simplified, robust, and power-efficient PARC engine that can be easily integrated in an e-nose system.

The emergence of neuromorphic methods provided a totally different outlook towards solving the artificial olfaction problem. The sparse spike-based data representation used in neuromorphic approaches was crucial for e-nose systems, as the volume of data generated could be minimized by encoding only useful information, enabling optimization of the processing [1,13,14]. Other advantages, such as low-power implementation and rapid processing of sparse data through spiking neural networks (SNNs) and bio-inspired learning algorithms, were vital for the development of efficient and robust artificial olfactory systems. The fully-integrated olfactory chip proposed by Koickal et al. in [15] was one of the first neuromorphic olfactory system implementations. Comprising a chemosensor array, a signal conditioning circuitry, and an SNN with bio-inspired learning capabilities, the proposed system emulated the sensing, transformation, and association functionalities of the biological counterpart. Although further research into overcoming the limitations of analogue design and real-world applications of this study was never reported, this groundbreaking work paved the path for future studies in neuromorphic olfaction.

Other noteworthy studies in neuromorphic olfaction include the rank-order-based latency coding [16,17], hardware-based olfactory models based on the antennal lobe of fruit fly [18,19,20], a VLSI implementation of an SNN based on the neurophysiological architecture of a rodent olfactory bulb [21], hardware implementation of the olfactory bulb model [22], a classifier using a convolutional spiking neural network [23], a 3D SNN reservoir-based classifier for odor recognition [24], and the columnar olfactory bulb model inspired by the glomerular layer of the mammalian olfactory pathway that was recently extended for its implementation on Loihi, Intel’s neuromorphic research chip [14,25]. However, most of the research in neuromorphic olfaction, such as [15,21,26,27,28,29,30], is more driven towards implementing a high level of bio-realism to emulate the biological olfactory pathway, which results in impractical models with limited scope for real-world applications [5]. Review articles [1,4,5,6] present a comprehensive survey on the development, application, and current limitations of neuromorphic olfactory systems.

Although application of neuromorphic methods and SNNs for artificial olfactory systems has begun to show promise, only a small number of studies, such as [12,14,21,24,31,32], have been able to deploy these bio-inspired models on an application-ready neuromorphic platform in a realistic field setting. In the work presented in this paper, we extend our previously reported neuromorphic encoding and SNN-based classification approach to include performance parameters when deployed on Akida neuromorphic hardware [12]. The significance of this work is two-fold: Firstly, the neuromorphic processing model for olfactory data hypothesized in [12] is proven by applying the model on a real-world dataset collected to identify eight types of malts. Secondly, the proposed neuromorphic model establishes a general platform for encoding and classifying e-nose data, where all of these functions can be mapped on the Akida neuromorphic hardware to leverage the ultra-low-power and high-performance capabilities for simplified integration in a portable e-nose system.

Studies based on implementation of traditional methods for evaluating malt aromas to identify malt types have shown them to be time-consuming and requiring use of costly equipment and trained personnel [33,34]. Accomplishing this task using a non-invasive electronic nose (e-nose) system may be of great interest within the brewing industry because malts, as one of the vital raw materials, significantly impact the beer quality and the brewing process [35]. However, achieving this presents a nontrivial classification task because, as is the case with most aromatic compounds, the instrumental odor characteristics of a malt sample may overlap even if their aroma profiles may seem different for human olfaction [36,37]. Therefore, this study aims to implement bioinspired data-encoding and classification techniques on olfactory data obtained using a commercial e-nose system and the Akida Spiking Neural Network (SNN) architecture.

## 2. Materials and Methods

### 2.1. Sample Preparation

The preparation of samples and experimental protocols were based on previous machine olfaction-based studies that included experiments with grains [38,39,40,41] and beer [42,43]. This study used eight types of malt samples obtained from Pilot Malting Australia. The classes of malts and their flavor profiles, as described in [36,44,45,46,47,48], are listed in Table 1. Samples were prepared using 100 g of each malt type transferred to a 250 mL sterile and borosilicate glass flask. The samples were sealed tightly with two layers of paraffin film and stored at room temperature to prevent the loss of volatiles and odor characteristics. Before exposure to the e-nose system, the samples were heated at 25 °C using a digital hotplate with frequent perturbation to ensure that the malts were evenly heated. The paraffin films were punched with holes to prevent moisture accumulation within the flask, and the perturbation continued until a thermal equilibrium was achieved. This process allowed the release of aromatic volatiles, which mainly include aliphatic alcohols, aldehydes, ketones, pyrroles, furans, and pyrazines [49], from the malt samples without a significant increase in relative humidity that would affect the headspace analysis. A total of eight samples, corresponding to each type of malt, were prepared for the experiment.

### 2.2. Electronic Nose System

A commercially available Cyranose-320™ e-nose (Sensigent, Pasadena, CA, USA) was used to obtain the aroma patterns from the headspace of the malt samples. The portable e-nose system incorporates a sensor array consisting of 32 nanocomposite sensors, where each sensor exhibits cross-sensitivity towards specific chemical or aromatic volatile compounds [50]. The e-nose system is exposed to these aromatic compounds through a delivery system where the chemical interaction between the sensing element and the volatiles results in a change in electrical resistance. This change in resistance is proportional to the amount of chemical absorbed by the conducting polymer on the sensing surface. The resulting signal is a change in resistance in a sensing element for the time interval during which it is exposed to the chemical vapors. The raw data acquired consists of changes in resistance in each sensor array element, producing a distribution pattern or a smell-print that can be used to identify the VOC mixture using pattern-recognition techniques. In the study described in this paper, it was observed that four sensors (sensors 5, 6, 23, and 31) were sensitive to polar compounds, such as water vapor due to moisture present in the headspace due to the heating of the malt samples. As a result, data from sensors 5, 6, 23, and 31 was not acquired during the experiments, and the experiments overall resulted in a 28-dimensional e-nose response.

### 2.3. Sampling Protocol

**Figure 1:**
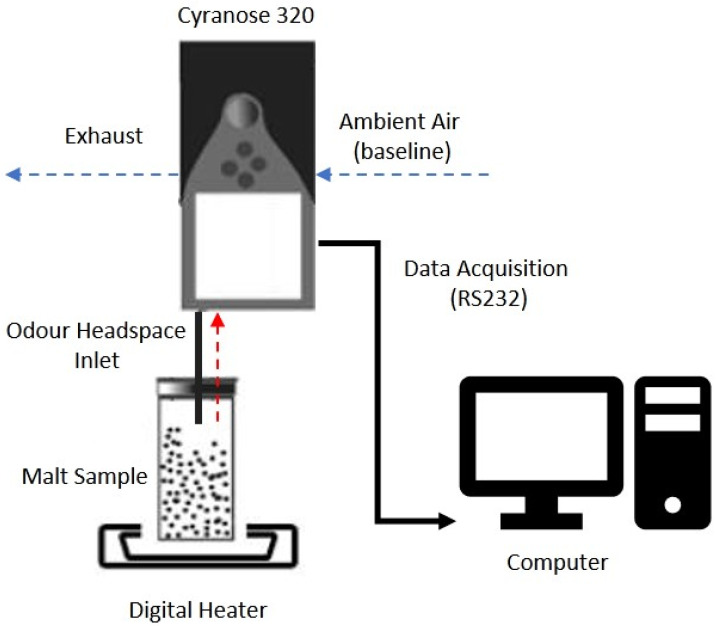
Experimental setup for headspace analysis of malt samples using Cyranose 320™.

The VOCs were measured using the experimental setup shown in Figure 1. Although the experiments were carried out in a fume cupboard to avoid interference from contaminants such as dust, ambient air was used for the baseline so as to replicate a real-world application where ideal lab conditions and zero-grade dry air for the baseline may not be available. Sensigent’s PCNose software was used for data acquisition, and the raw resistance change data was exported to a CSV file. As reheating of the malt sample after initial thermal equilibrium was achieved could potentially change its physical characteristics and adversely affect the experiments, data samples were recorded as consecutive sensor response measurements until the thermal equilibrium could be maintained. In total, nine replicates of measurements were recorded for each malt sample, resulting in a dataset of 72 files with eight classes. Another set of experiments producing three additional replicates per class was carried out under similar laboratory conditions. This dataset, consisting of 24 files, was used to validate the classifier’s generalization for inferences of previously unseen data.

Before the experiments, the e-nose system was purged with ambient air for six minutes to obtain a steady baseline. For the e-nose analysis, the sample headspace was analyzed for a total of 90 s. This included 15 s of baseline, 50 s for sample intake, and 25 s for snout removal and baseline purge. The substrate temperature was set to 37 °C and the pump speeds for each sampling stage were set as per the manufacturer’s recommendation [50], and the sampling frequency was set to 1 Hz. Table 2 shows the sampling parameters used to record responses from the e-nose system.

### 2.4. Signal Conditioning and Pre-Processing

**Figure 2:**
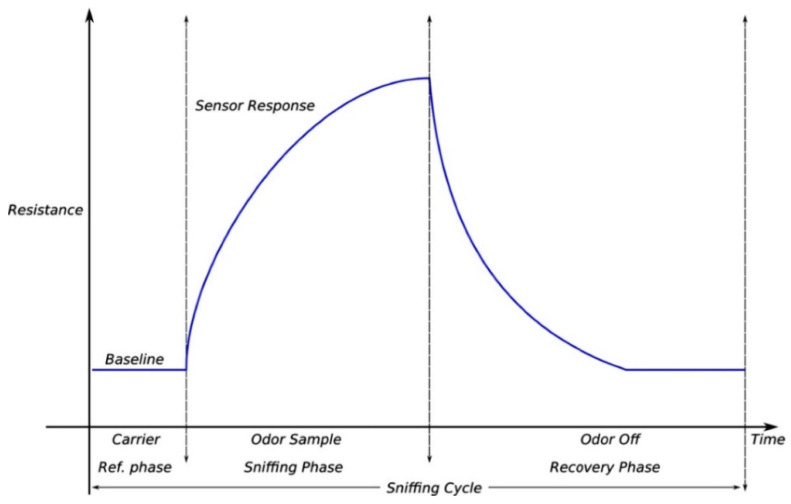
Typical response signal of an electronic nose sensor for a sniffing cycle (adapted from [51]).

The odor data acquired in the form of relative resistance signals was first visually analyzed using the PCNose tool, which is Sensigent’s interfacing and data acquisition software for the Cyranose-320™ e-nose system. A typical e-nose response has three key components: a baseline response during the reference phase, a response curve and steady response during the exposure/sniffing phase, and a transition back to the baseline during the recovery phase (shown in Figure 2) [51].

In order to accomplish the identification of aromatic compounds through pattern recognition of e-nose responses, raw sensor responses have to be conditioned to mitigate the effects of noise and differences in resistance ranges of the sensors that can influence the outcomes of the classification process [7,11]. Noise in the sensor responses was mitigated by implementing a rolling mean smoothing technique, and the signals were normalized by fractional manipulation during which the baseline is subtracted from the signal and divided by the minimum and maximum resistance to generate dimensionless and normalized responses on a unified scale between 0 and 1. In general, normalization using linear scaling was used over other methods in order to avoid computationally expensive operations during the pre-processing stage.

Mathematically, the normalization process can be expressed as:

$$\left|R_{norm}\left(x\right)\right|=\frac{R_{i}-R_{0}}{R_{\max\left(x\right)}-R_{\min\left(x\right)}}$$

where $R_{norm}\left(x\right)$ is the absolute value of normalized relative resistance for sensor x, $R_{0}$ is the baseline response of sensor x, $R_{i}$ is the measured resistance of sensor x at instance i, and $R_{\min\left(x\right)}$ and $R_{\max\left(x\right)}$ are the minimum and maximum resistance of sensor x for that sample.

Although the dataset was limited in terms of the number of samples and classes, each sample is highly multidimensional as responses from 28 sensors are acquired. Despite the fact that each sensing element responds differently to the aromatic compounds, the distinctive information observed in the dataset is limited as the sensor responses follow a typical trend of baseline response followed by an increase or decrease in resistance to a steady-state response when exposed to the malt sample and back to baseline during the recovery phase. As a result, except for the slope of the sensor responses, most of the time-points represent a steady-state feature that may not suffice for classification, especially for a highly multivariate dataset.

Another feature set based on enhancing inter-class discrimination was extracted to overcome the limitations of relative resistance features. In this case, the mean of the baseline was subtracted from the signal, and the data was normalized using the min–max values recorded for each sensor across all samples and classes. This global normalization process can be modelled as:

$$\left|R_{norm}\left(x\right)\right|=\frac{R_{i}-R_{baseline\left(avg\right)}}{R_{global\;max\left(x\right)}-R_{global\;min\left(x\right)}}$$

where $R_{norm}\left(x\right)$ is the absolute value of normalized resistance response for sensor x, $R_{i}$ is the measured resistance of sensor x at instance i, $R_{baseline\left(avg\right)}$ is the average of sensor x’s baseline response, and $R_{global\;max\left(x\right)}$ and $R_{global\;min\left(x\right)}$ are the global maximum and minimum resistances for sensor x observed across all samples and classes.

**Figure 3:**
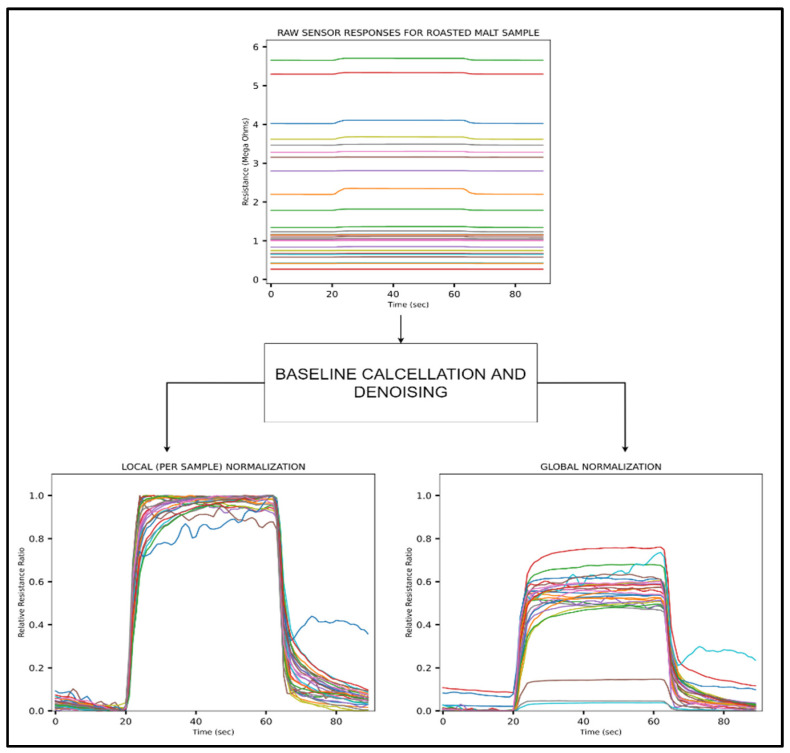
The transformation of raw sensor responses for a roasted malt sample into useful feature sets using global and local normalization.

The implementation of global normalization highlighted the descriptive information regarding the sensor responses with respect to each class by enhancing their inter-class features. This unique information can be used to distinguish sensor responses more effectively, which boosts classification performance. The pre-processing and conditioning stage is illustrated in Figure 3, which shows the transformation of the raw signal into features that were used for encoding and classification.

### 2.5. Data-to-Event Encoding Using AERO

One of the key aspects of implementing a neuromorphic approach for a sensing application is the sparse representation of data using a spike-based format that enables rapid processing with minimal power consumption [52]. Although the encoding of data in a spiking format can be achieved using several bioinspired algorithms, such as step forward (SF) thresholding or Ben’s spiker algorithm (BSA) [53], address event representation (AER) [54] has become a de facto standard within the neuromorphic domain [55]. Based on the abstraction of the pulse-based neurobiological communication code found in living organisms, AER is an ideal interface for communicating temporal information in an event-based sparse format from multiple sources using a narrow channel [56].

First conceptualized during the development of the dynamic vision sensor (DVS), the AER protocol’s ternary data format for vision applications is used to encode *X*-axis and *Y*-axis coordinates of a pixel and ON or OFF spikes that are generated using a thresholding method to represent luminosity changes [4,57]. Following the successful implementation of AER for neuromorphic vision sensors, the AER protocol has been extended for several other neuromorphic systems, such as tactile [58,59] and auditory sensing [60], along with event-driven processing in neuromorphic hardware implementations [52,61,62].

The data-to-event transformation approach used in this work was abstracted from our previously developed AER for olfaction (AERO) encoder [12]. This approach is based on quantizing the normalized sensor responses to encode signal amplitude levels of each sensor within the AER data structure. AERO generates events at each timepoint and translates sensor responses into the AER-based spiking data format to encode the timestamp, the amplitude level of the signal, and the sensor ID information. Similar to one-hot encoding [63], the quantization of the signal amplitude creates time-based bins that are used by the SNN to learn from the non-zero bins and classify the sensor responses.

**Figure 4:**
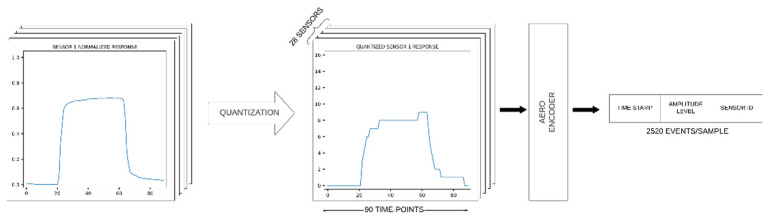
An instance of the quantized signal for sensor 3 from the roasted malt sample.

Based on the number of bits selected for quantization, the signal amplitude is partitioned into $2^{n}$ levels, where *n* is the number of bits used. The quantization levels of signal amplitude are crucial to preserve the features that can significantly influence the learning and classification capabilities of the SNN. Typically, the number of bits selected for quantization determines whether the time-based bins formed are fine- or coarse-grained, which directly impacts the SNN’s ability to generalize the odor classes based on the class-specific features it has learnt. This process of encoding continuous e-nose sensor responses into sparse AER-based events implemented through AERO is illustrated in Figure 4 as a conceptual block diagram.

### 2.6. Akida Neuromorphic Framework and Network Architecture

Spiking neural networks are a particular class of artificial neural networks (ANNs) that incorporate biological processing principles where neurons process and propagate information in the form of sparse action potential-like representations, also known as spikes. The Akida neuromorphic framework by Brainchip implements these core concepts in the form of a digital neuromorphic system-on-a-chip (NSoC) [64] and the Akida Execution Engine (AEE), a Python-based chip emulator and key component of the Akida MetaTF ML framework (link—https://doc.brainchipinc.com accessed on 15 November 2021) for development and simulation of the behavior of the SNNs supported by the event domain neural processor.

The Akida SNN implements a simplistic yet effective integrate-and-fire neuron model where a summation operation of input spikes is performed to simulate the membrane potential of the neuron and causes the neuron to fire if this potential is higher than a predetermined threshold. One of the key features of this neuromorphic framework is the binary implementation of synaptic weights and activation. This significantly reduces the computational overhead, resulting in a low-power rapid processing architecture [65].

**Figure 5:**
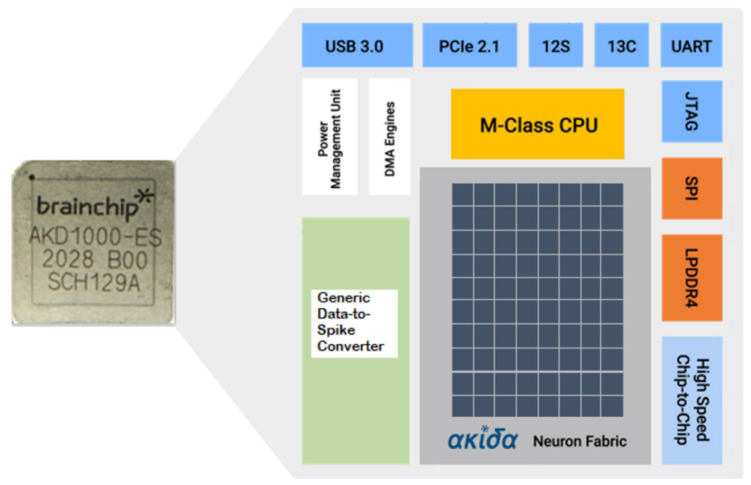
Block diagram of the Akida NsoC consisting of an event encoder, onboard CPU, and the Akida Neuron Fabric. Published with copyright permission from Brainchip Inc.

The study described in this paper takes advantage of the fact that SNN models developed using the Akida MetaTF framework can be seamlessly deployed on the Akida NsoC, allowing the classifier to run on low-power neuromorphic hardware with support for edge learning. Additionally, the on-chip processor and data-to-spike converter within the Akida NsoC architecture (shown in Figure 5) enables onboard signal pre-processing and event generation, thus eliminating the requirement of a PC for interfacing with the e-nose system.

The neuromorphic classifier proposed in this work is based on a feed-forward two-layer network architecture that comprises an input layer that receives AER-based spiking input and a fully connected layer for processing. The input dimensions, such as the number of timepoints (input width), activation levels (input height), and the number of features (number of sensors), are defined in the input layer. The event-based data generated by the AERO encoder is received by the input layer and propagated as spikes to the subsequent fully connected processing layer. This layer is responsible for learning and classification tasks. Several parameters—such as connectivity of neurons, the total number of neurons, minimum plasticity, and learning competition—are defined in this layer, which control the learning and classification performance of the model.

## 3. Results and Discussion

### 3.1. Classifier Training: Learning Using STDP

Learning in the SNN-classifier is implemented using the Akida built-in learning algorithm based on the bioinspired spike-time dependent plasticity (STDP) learning approach with modifications for efficient implementation on low bit-width architectures (refer to [66]). In this unsupervised learning approach, the neurons learn to respond to particular features that are found to repeat over multiple input samples by reinforcing the synapses that match an activation pattern [64]. The synaptic connectivity of the neurons within the network undergoes weight changes to establish a correlation with repeating temporal patterns, and the competition between neurons ensures that they each learn different features.

The quantization of the signal during the data-to-event encoding plays an important role in the learning process as the discretized sensor responses are distributed in time-based bins, similar to one-hot encoding, and the network learns the signal characteristics and odor-specific features from non-zero-valued bins. In this case, the level of quantization controls the specificity and generalization of the signal that the network learns over successive presentation of the e-nose data. A 4-bit discretization that partitions the amplitude of the signal into 16 activation levels was selected for this application based on the overall classification performance of the network achieved with minimum use of neural resources.

**Figure 6:**
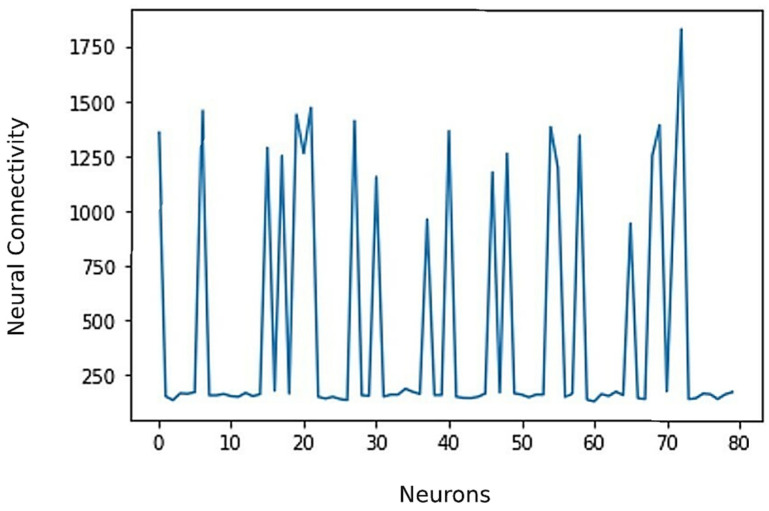
Changes in neuron weights after the training phase. In this case, 19 neurons among the neuron population of 80 neurons have learnt to identify key patterns within the sensor responses for eight classes of malts resulting in synaptic changes.

Training the SNN model was based on one-shot learning where the SNN learns repeating temporal patterns through a single feed-forward propagation of event-based data. This approach is much faster than typical deep learning gradient-based training that requires multiple iterations for network convergence and to minimize the error function. Training and testing of the SNN-based classifier for all eight classes of malts was implemented for both of the relative resistance features (local and global) that were extracted during the pre-processing stage. In each case, a randomly allocated combination of six files per sample (70%) were used for training the classifier model, and the remaining three files (30%) were used for testing. The resultant connectivity weights within the neuron population after the learning phase for locally normalized relative resistance features are shown in Figure 6.

### 3.2. Classification Performance

The classification within the SNN is based on a winner-takes-all (WTA) logic [67], where the class label of the neuron with the highest activation level among the population is allocated to the presented data. The accuracy of the classifier is determined by comparing the predicted class label to the true class label for the validation data. The experiments for classification of malts using the SNN model were conducted for both of the extracted features, locally normalized relative resistance and relative resistance normalized using global min–max.

An optimization process based on differential evolution [68] was implemented to determine a configuration for key parameters of the network. These include the minimum plasticity, plasticity decay, and learning competition, which have a significant influence on the classification performance of the SNN model. The optimum values for the network parameters were derived using a fitness function based on maximizing the stable classification accuracy of the SNN model. Certain parameters—such as the number of neurons per class and the connectivity of neurons (number of weights per neuron)—largely depend on the number of samples within a class, the number of sensors (dimensions of the data) employed, and the number of timepoints used for classification. The initial plasticity parameter was set to the maximum during the network initialization and gradually decreased based on the neuron activations and learning. Table 3 lists the network parameters, a short description of their functionality, their bounds used for the optimization process, and the optimum values for each parameter.

The classification performance of the network was determined using a stratified five-fold cross-validation. For the first scenario using the locally normalized relative resistance feature, the SNN model provided a classification performance of 90.83% with a variance of ±4.083%. The classification performance of the SNN model for the second scenario using relative resistance normalized using global min–max increased by 6.25%. In this case, the five-fold cross-validation accuracy of the classifier was found to be 97.08%, with a variance of ±2.08%. For each scenario, the processing latency for the emulated learning and recognition tasks on a standard PC with an i5 CPU, including the data-to-event encoding and other software-based latencies due to looping and control structures, was found to be between 1.5 and 2 s.

In order to evaluate the efficiency and accuracy of the SNN-based classifier in regard to the overall classification performance, we compared the obtained results with statistical machine learning tools. As most of the statistical classification methods are based on single vector inputs [7,11,13], the temporal data was reduced to three static features: maximum resistance change, area under the curve, and the slope of the sensor response during the sniffing phase of the sampling. Statistical machine learning algorithms generally do not perform well for highly multidimensional datasets [1,5,24]. Hence, principal component analysis (PCA) was used for dimensionality reduction and the dataset was reduced to three key components based on maximum explained variance. The comparison of classification accuracy and latency to train and classify the dataset based on a 70:30 train:test split and five-fold cross-validation is shown in Table 4 below.

**Figure 7:**
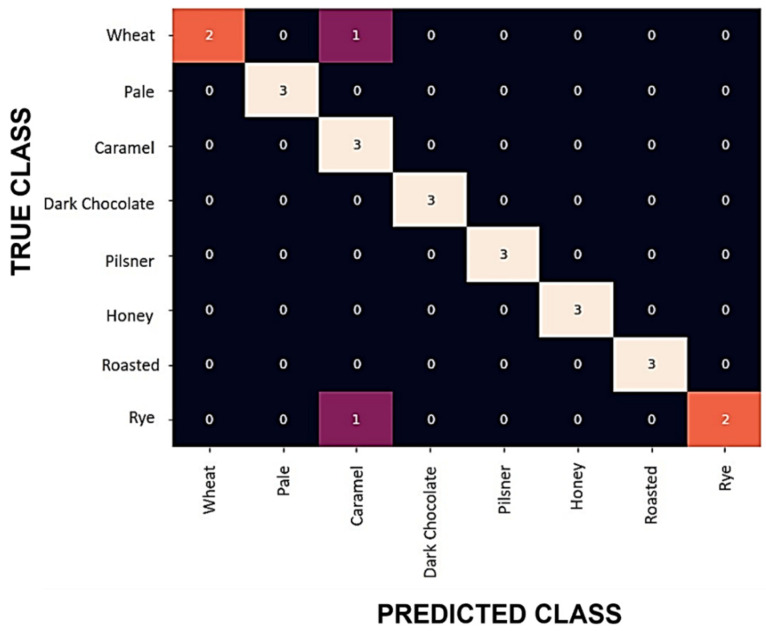
Confusion matrix for the classification of the ‘inference-only’ dataset consisting of 24 samples. Only two misclassifications were observed, one of which can be attributed to overlapping aroma profiles of wheat and caramel malts.

In order to validate the classifier performance, the SNN model was exposed to an entirely unseen dataset. This phase of the work used the secondary dataset, consisting of 24 files. This test was crucial to evaluate the generalization ability of the classifier model and eliminate the effects of inadvertent overfitting resulting from multiple uses of data during the model development. Applying the SNN model to this dataset resulted in 91.66% accuracy for the relative resistance features using global normalization. A confusion matrix of the classification result is shown in Figure 7.

As SNN models designed using the Akida MetaTF framework can be seamlessly deployed on the Akida NsoC, the SNN-based classifier proposed in this study was implemented on the Akida neuromorphic hardware to validate the performance parameters. All functionalities of the proposed pattern recognition engine, including pre-processing, AERO encoder, and the SNN-based classifier, were mapped onto the neuromorphic hardware platform. As anticipated, the classification performance of the SNN model when implemented on the hardware was similar to the results obtained using the software-based chip emulator. The classification latency for a trained SNN model in an inference mode was recorded to be 0.6 ms per inference. The dynamic power consumption of the SNN-based classifier when implemented on the neuromorphic hardware was less than 1 mW. The overall classification results, on both the Python-based emulator and the neuromorphic hardware, confirm that the proposed neuromorphic framework can be efficiently integrated as a pattern recognition engine in a portable artificial olfactory system operating under strict power constraints to deliver highly accurate classification in real time.

## 4. Conclusions

This study presents the implementation of a neuromorphic approach towards the encoding and classification of electronic nose data. The proposed approach was used to identify eight classes of malts and has potential as an application for quality control in the brewing industry. Experiments were conducted using a commercial e-nose system to record a dataset consisting of time-varying information of sensor responses when exposed to different malts under semi-laboratory conditions. The classifier proposed in this study utilized the combination of the Akida SNN and the AERO encoder, a neuromorphic approach that has previously delivered highly accurate results on a benchmark machine olfaction dataset [12]. The proposed method successfully classified the dataset with an accuracy of 97.08% and a maximum processing latency of 0.4 ms per inference when deployed on the Akida neuromorphic hardware. A secondary dataset that was used to validate the classifier model in an ‘inference-only’ mode was classified with an accuracy of 91.66%. These results could potentially be further improved by refinements to pre-processing that can enhance informative independent components for malt classes that are misclassified.

Based on these results, we can conclude that the classifier model implemented using Akida SNN in conjunction with the AERO encoder provides a promising platform for odor recognition systems. An application targeted towards the identification of malts based on their aroma profile, generally considered a nontrivial classification task using traditional machine learning algorithms, was successfully demonstrated in this work with a classification accuracy greater than 90% under different scenarios. The developed model can be deployed on the Akida NsoC, thus enabling the integration of a bio-inspired classifier model within a commercial e-nose system. A comparative analysis of the proposed approach with statistical machine learning classifiers shows that the SNN-based classifier outperforms the statistical algorithms by a significant margin for both accuracy and processing latency. A performance-based comparison of the neuromorphic model proposed in this work with other neuromorphic olfactory approaches, such as [13,14,26,27,69,70], could not be established as their inherent structures, including spike encoding schemes, neuron models, SNN architectures, and implementation of learning algorithms, vary vastly. The proposed methodology, however, does not require a graphic processing unit (GPU)-based model simulation, unlike in [13], or a complex bio-realistic model, as used in [14]. Furthermore, the SNN-based classifier can be entirely mapped on a single neural processing unit core, as opposed to multiple cores used in [14], leading to a low-power and low-latency implementation.

The application of such real-time and highly accurate e-nose systems can be extended to fields such as food technology, the brewing and wine industries, and biosecurity. Future research in this domain will focus on encoding parameters such as rank-order code within the AERO events to analyze its impact on classification performance.

## Figures and Tables

**Table 1:** Types of malts used in this study and their flavor descriptors.

Malt Type	Flavor Descriptors
Wheat	Clove-like and banana notes with malty sweetness
Pale	Sweet and slightly biscuity
Caramel	Sweet, honey-like with slight roasty/toastiness
Dark chocolate	Rich roasted, coffee, and cocoa
Pilsner	Mild sweetness with straw/grassy notes
Honey	Subtle honey and bread flavors
Roasted	Coffee, intense bitter, and roasty notes
Rye	Roasty and spicy notes

**Table 2:** Signal acquisition parameters for the e-nose system.

Parameter	Time	Pump Speed
Baseline correction	15 s	Medium (120 cc/min)
Sample draw-in	50 s	High (180 cc/min)
Snout removal	5 s	
Purge (air intake)	20 s	High (180 cc/min)
Substrate heater temperature	37 °C	

**Table 3:** SNN parameters with a description of their functionality, their max–min bounds used for the optimization, and the optimum value of the parameter obtained using grid-search.

Network Parameters	Parameter Description	Bounds	Optimum Value
Number of neurons per class	Number of neurons representing each class	1–30	10
Number of weights per neuron	Number of active connections for each neuron	1 to 2880 (max bound is derived from 2 × number of timepoints × quantization levels)	1795
Initial plasticity	Controls weight changes when learning occurs	0.75–1.00	0.84
Learning competition	Controls competition between neurons	0.1–0.75	0.48
Minimum plasticity	Minimum level to which connectivity among the neurons will decay	0.1–0.50	0.21
Plastic decay	Decay of weight connections with each learning step	0.1–0.50	0.27

**Table 4:** Comparative analysis of the proposed approach and other statistical machine learning classifiers’ classification performance.

Method	Classification Accuracy	Execution Time
Akida SNN (this work)	97%	1.85 s
Linear Discriminant Analysis	84%	33 s
Support Vector Machine	89%	22 s
K-Nearest Neighbor (weighted)	73%	14 s
